# Alternative Splice Variants in TIM Barrel Proteins from Human Genome Correlate with the Structural and Evolutionary Modularity of this Versatile Protein Fold

**DOI:** 10.1371/journal.pone.0070582

**Published:** 2013-08-12

**Authors:** Adrián Ochoa-Leyva, Gabriela Montero-Morán, Gloria Saab-Rincón, Luis G. Brieba, Xavier Soberón

**Affiliations:** 1 Instituto Nacional de Medicina Genómica (INMEGEN), México City, México; 2 Laboratorio Nacional de Genómica para la Biodiversidad, Centro de Investigación y de Estudios Avanzados del Instituto Politécnico Nacional, Irapuato, Guanajuato, México; 3 Departamento de Ingeniería Celular y Biocatálisis, Instituto de Biotecnología, Universidad Nacional Autónoma de México, Cuernavaca, Morelos, México; NIGMS, NIH, United States of America

## Abstract

After the surprisingly low number of genes identified in the human genome, alternative splicing emerged as a major mechanism to generate protein diversity in higher eukaryotes. However, it is still not known if its prevalence along the genome evolution has contributed to the overall functional protein diversity or if it simply reflects splicing noise. The (βα)_8_ barrel or TIM barrel is one of the most frequent, versatile, and ancient fold encountered among enzymes. Here, we analyze the structural modifications present in TIM barrel proteins from the human genome product of alternative splicing events. We found that 87% of all splicing events involved deletions; most of these events resulted in protein fragments that corresponded to the (βα)_2_, (βα)_4_, (βα)_5_, (βα)_6_, and (βα)_7_ subdomains of TIM barrels. Because approximately 7% of all the splicing events involved internal β-strand substitutions, we decided, based on the genomic data, to design β-strand and α-helix substitutions in a well-studied TIM barrel enzyme. The biochemical characterization of one of the chimeric variants suggests that some of the splice variants in the human genome with β-strand substitutions may be evolving novel functions via either the oligomeric state or substrate specificity. We provide results of how the splice variants represent subdomains that correlate with the independently folding and evolving structural units previously reported. This work is the first to observe a link between the structural features of the barrel and a recurrent genetic mechanism. Our results suggest that it is reasonable to expect that a sizeable fraction of splice variants found in the human genome represent structurally viable functional proteins. Our data provide additional support for the hypothesis of the origin of the TIM barrel fold through the assembly of smaller subdomains. We suggest a model of how nature explores new proteins through alternative splicing as a mechanism to diversify the proteins encoded in the human genome.

## Introduction

Protein functions and folds evolve through changes in the amino acid sequence such as insertions, deletions and substitutions [1]. Furthermore, structural and functional diversity can be generated through rearrangements of protein modules taken from pre-existing archetypical domain repertoires. Sequence diversity can be generated by both homologous and nonhomologous recombination processes. Nonhomologous recombination has been theorized to be the most effective mechanism at enabling new structures and functions to emerge during protein evolution [1], suggesting that combinatorial assembly and modular substitutions between segments of unrelated proteins play an important role in evolution [2]. In addition, alternative splicing, which combines small gene segments and occurs as a normal process in eukaryotes, greatly increases the sequence diversity of proteins encoded in a genome [3]. Through alternative splicing, a single gene may encode multiple proteins. For instance, it is estimated that up to 95% of multi-exon genes are alternatively spliced in humans [4]. Alternative splicing is a regulatory process working independently of transcriptional regulation and provides an additional control of tissue-specific gene expression [5]–[8]. Furthermore, alternative splicing can also influence the mechanisms of enzyme activity regulation, protein oligomerization and protein-protein interactions [9]. However, there is still limited knowledge derived from experimental data regarding the structural and functional consequences of alterations in proteins resulting from alternative splicing events and their role in expanding the functionality of a eukaryotic proteome [9]–[11].

The large increase of available RNA sequences for the human genome, which were exponentially expanded by the incursion of Next-Generation Sequencing technologies, has opened the possibility for analyses of the role that insertion, deletion, and substitution play as elements that generate protein diversity as a consequence of the alternative splicing process [12], [13]. The human genome is one of the most studied genomes of all the model organisms. However, the current knowledge of alternatively spliced variants is derived mainly from mRNA transcripts, and very little is known about their fate as proteins with specific tertiary structures [3], [11].

The exon shuffling theory of genes suggests that proteins acquired their functional diversity by combining gene segments encoded by ancient exons in the early stages of protein evolution [14], [15]. Consistent with this notion, several analyses of alternative splice variants have demonstrated a strong correlation between DNA from exonic regions and structural and functional motifs of proteins [10], [16]. The recombination of such subunits could lead to the diversification of domain architecture, generating proteins from which new folds and functions could have emerged. On a scale smaller than the large multi-domain protein complexes, folding and evolving structural modules can also be defined within individual proteins. An example of the latter is the (βα)_8_ barrel or TIM barrel, which constitutes one of the most frequent, versatile, and ancient fold encountered among the known enzymes [17]–[19]. The canonical topology of this fold consists of eight repeats of (βα) modules. The β-strand and the α-helix within a given module are linked by a βα-loop, and the α-helix of one module is linked to the β-strand of other module by an αβ-loop. The eight β-strands form the central barrel, which is surrounded by eight α-helices. Different structural analyses and protein fragmentation experiments have suggested that (βα)_8_ barrel proteins can be divided into several subdomains that can be related to independently folding and evolving structural units [20]–[23]. The barrel subdomains may comprise different numbers of (βα) modules, i.e., the (βα)_2_, (βα)_4_ and (βα)_6_ subdomains. Substitutions of (βα) modules and βα-loops have been shown to cause no significant disruption of the structure in some members of the fold [24]–[26]. Segments small as a βα-loop can be considered to be a functional protein module that is able to play a key role in the divergence of enzyme functions [25], [27]. The high level of internal symmetry within the (βα)_8_ barrel has led to the speculation that it evolved through duplication and fusion of smaller barrel subdomains. Interestingly, when different part-barrel subdomains were co-expressed *in vivo* and *in vitro*, both subdomains were reassembled noncovalently to form the catalytically active (βα)_8_ barrel [23], similar to “Lego” pieces. Additionally, there is evidence that several (βα)_8_ barrels fold via smaller pre-folded substructures, comprising part-barrel subdomains [18], [28], [29]. Together, these studies suggest that many (βα)_8_ barrels may have evolved from ancestors composed of different subdomains such as a stable three-quarter barrels or half barrels. However, a genetic mechanism for a natural process of the reassembly and evolution of the barrel subdomains has not been described.

In this work, we analyze the structural changes that occur in (βα)_8_ barrel proteins derived from the human genome as a consequence of alternative splicing by using experimental and bioinformatics analyses. From a protein engineering perspective, each alternative splice variant can be viewed as an engineered protein product by sequence insertion, deletion, or substitution. We provide examples of how the resulting splice variants may represent structurally viable subdomains of the (βα)_8_ barrels, and we explain these links by a common genetic mechanism. Furthermore, we discuss how the alternative splicing process can provide insights regarding the evolution of this fold and its role in the expansion of human proteome diversity. In the absence of previous information about the functional and structural consequences of β-strand or α-helix substitution in protein members of the (βα)_8_ barrel, we designed protein engineering experiments using *E. coli* phosphoribosylanthranylate isomerase (TrpF) as a (βα)_8_ model enzyme. The experiments consisted of the substitution of one β-strand and one α-helix of the *E. coli* TrpF by gene segments derived from a different enzyme. Our analyses reinforces the notion that the (βα)_8_ barrel proteins can be made up of structural subdomains. We suggests a model of how the barrel subdomains can be rearranged to diversify the (βα)_8_ barrel proteins encoded in the human genome as opposed to just noise. Our model offers an explanation to the large diversity of proteins generated through alternative splicing.

## Results

### Identification of the (βα)_8_ barrel proteins encoded in the human genome

**Figure 1:**
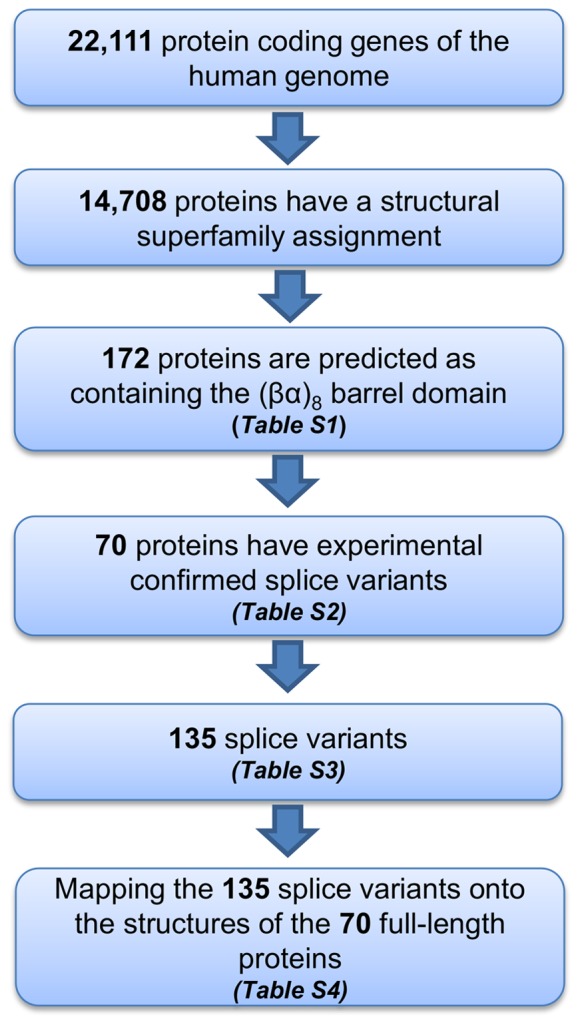
Pipeline of bioinformatics analysis to identify the (βα)_8_ barrel proteins with splice variants in the human genome. Summary of our bioinformatics data flow to extract the 135 experimentally confirmed (βα)_8_ barrel splice variants in the human genome.

First we retrieved all of the predicted proteins containing structural superfamily assignments from the human genome using Gene3D (Figure 1) [30]. From the 14,708 protein-coding genes of the human genome that have at least 1 CATH structural superfamily assignment, we retrieved 172 protein-coding genes containing a predicted (βα)_8_ barrel domain (Table S1). Genes were selected for further analysis if, according to the UniProtKB database, there is experimental evidence for proteins derived from alternative splicing or at least one mRNA with correct intron/exon boundaries [31]. Out of the 172 putative (βα)_8_ barrel proteins, 70 protein coding genes have experimentally confirmed splice variants (Table S2), resulting in a total of 135 splice variants (Table S3).

### Mapping of the splice variants onto the structures of the full-length proteins

Twenty-six of the 70 full-length proteins have a structure reported in the Protein Data Bank, and high-quality structural models were obtained for 44 additional proteins using ModBase (see Materials and Methods). To identify the structural changes as a consequence of the alternative splicing process, we mapped all of the 135 splice variants onto the structures of the 70 full-length proteins (Table S4). We inferred that the alternative splicing events directly affected the structure of the (βα)_8_ barrel for 67 splice variants (Table S5). This set of 67 splice variants represent a small subset of all possible for the (βα)_8_ barrels predicted for the human genome. However, it contains curated information on the experimental existence of the mRNA from the splice variants. In addition, the corresponding full-length proteins of this set contain protein members that represent 11 of the 17 CATH superfamilies found for all of the (βα)_8_ barrel proteins from the human genome (Table S6). The majority of the alternative splicing events fall into the loops or in the first residue of the β-strand or α-helix elements of the (βα)_8_ barrel structure. Interestingly, alternative splicing events fall in the middle of a β-strand or α-helix element only in 15% of cases. The fact that splicing events fall into non-structured regions of the (βα)_8_ barrel structure, suggest the presence of well-structured complete subdomains as exchanging units.

### Analysis of structural modifications of the (βα)_8_ barrel as a consequence of alternative splicing

**Figure 2:**
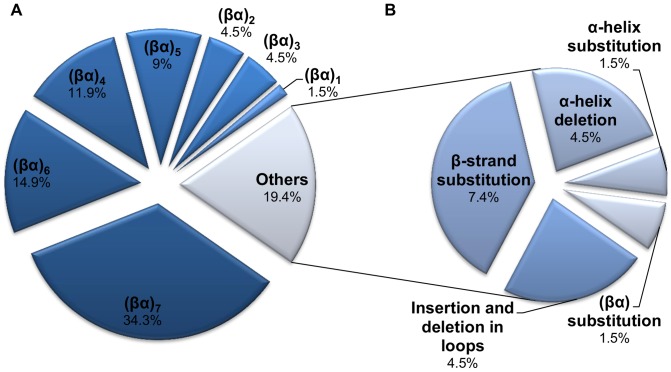
Subdomain distribution of 67 splice variants found in the (βα)_8_ barrel proteins from the human genome. The different (βα)_8_ barrel subdomains present in the splice variants are illustrated in a). The typical secondary-structure composition of the canonical (βα)_8_ barrel consists of eight repeats of βα modules. The secondary-structure composition of different (βα)_8_ barrel subdomains is described in A. The number of splice variants for each subdomain category is as follows: (βα)_1_ = 1, (βα)_2_ = 3, (βα)_3_ = 3, (βα)_4_ = 8, (βα)_5_ = 6, (βα)_6_ = 10, (βα)_7_ = 23, others  = 13. The subgroup of the 13 splice variants that cause structural modifications to the (βα)_8_ barrel proteins without altering their overall fold structure are illustrated in B. The number of splice variants for each category is as follows: β-strand substitution  = 5, α-helix deletion  = 3, insertion and deletion in loops  = 3, (βα) substitution  = 1, α-helix substitution  = 1. All the illustrated data were taken from Table S5.

We found that 54/67 splicing events involve sequence deletions in which a part of the full-length protein sequence was removed (Figure 2a). These results are in agreement with previous observations that demonstrated that splice variants with deletions are the most abundant of all splicing events [11].The majority of splicing events resulted in protein fragments corresponding to the (βα)_4_, (βα)_5_, (βα)_6_, and (βα)_7_ subdomains of the barrel (Figure 2a). The remaining 13 alternative splicing events resulted in (βα)_8_ barrel proteins with a loop insertion/deletion, β-strand, and (βα) substitution and α-helix deletion. Figure 2b shows the subset of the 13 alternative splice variants that was predicted to cause modifications to (βα)_8_ barrel proteins without altering their overall structure. Interestingly, β-strand substitutions that occur in the inner part of the barrel are the majority of the splicing events from this subgroup (Figure 2b) and constitute 7.4% of all splice variants (Figure 2a).

### Protein engineering experiments comparable to the structural changes of splice variants

The large data set of experiments with the (βα)_8_ barrel allows us to analyze if the structural changes found in the splice variants have been previously observed in protein engineering experiments and to explore its consequences on the structure-function of the (βα)_8_ barrel proteins (Table 1). To that end, we also examined the 67 splice variants for experimental functional annotations in the UniProtKB and we found them (either at protein level, protein solubility, and differential gene expression) for 17 splice variants (Table 2). Many of these splice variants containing barrel subdomains have been previously confirmed at the protein level and were found also to be differentially expressed in different human tissues (Table 2). Interestingly, the subdomain configurations found in the splice variants (Figure 2a) have been previously observed by protein engineering experiments as soluble and stable protein fragments or as structured folding intermediates in different (βα)_8_ barrel proteins (Table 1) [23], [28], [29], [36]. Proteins with a (βα)_7_ composition were the most abundant splice variants. Three of the four splice variants that contain the (βα)_7_ subdomain were previously reported as soluble proteins (Table 2) and this subdomain, which contains only seven β-strands, has been reported as a natural deviation from the canonical topology occurring in certain cellulases, flavoproteins, and in different members of the nicotinate/quinolinate PRTase C-terminal domain-like superfamily (Table 1) [38]–[40]. We found that splice variant Q00722–2 has a deletion of four residues in a βα loop and has been reported as a soluble and active enzyme (Table 2). Sequence insertion/deletion and substitution at βα-loops have been shown to have no significant disruption of the structure and function in some members of the fold (Table 1) [24]–[26].

**Table 1:** (βα)_8_ subdomains and barrel modifications reported by protein engineering experiments.

(βα)_8_ subdomain and barrel modification	Reported as stable and folded structure	Reported as structured folding intermediate	Catalytic activity	Refs.
(βα)_2_	Yes	Yes	Inactive	[21], [32]
(βα)_3_	No	Yes	Inactive	[29], [33]
(βα)_4_	Yes	Yes	Inactive	[23], [34]
(βα)_5_	Yes	Yes	Inactive	[28], [29]
(βα)_6_	Yes	Yes	Inactive	[22], [29], [35]–[37]
(βα)_7_	Yes	No	Active	[38]–[40]
(βα)_8_ with loop insertion, deletion or substitution	Yes	NA	Active	[25]–[27], [41], [42]
(βα)_8_ with βα substitution	Yes	NA	Active	[24], [32]
**(βα)_8_ with β-strand substitution**	Yes	NA	Active	**This work**
**(βα)_8_ with α-helix substitution**	Yes	NA	Active	**This work**

NA: Not assigned because the corresponding structure forms the overall barrel domain.

**Table 2:** Functional annotations of human splice variants.

Barrel subdomain	Gene name	Splice variant	Detected at protein level	Solubility	Catalytic activity	Tissue-specific gene expression	Refs.
(βα)_1_	HYAL3_HUMAN	O43820–4	Yes^a^	ND	Inactive	Yes	[43]
(βα)_2_	HYAL3_HUMAN	O43820–3	Yes^a^	ND	Inactive	Yes	[43]
	HYAL1_HUMAN	Q12794–5	Yes^a^	ND	Inactive	Yes	[43]
(βα)_3_	ADC_HUMAN	Q96A70–5	Yes^a^	ND	Inactive	Yes	[44]
(βα)_4_	HYAL1_HUMAN	Q12794–3	Yes^a^	ND	Active	Yes	[43]
	BGAL_HUMAN	P16278–2	Yes	Soluble	Inactive	Yes	[45]
	CHIA_HUMAN	Q9BZP6–3	ND	ND	ND	Yes	[46]
(βα)_5_	HYAL1_HUMAN	Q12794–4	Yes^a^	ND	Inactive	Yes	[43]
(βα)_6_	HMGCL_HUMAN	P35914–2	ND	ND	ND	Yes	[47]
(βα)_7_	HYAL3_HUMAN	O43820–2	Yes^a^	ND	Inactive	Yes	[43]
	HYAL1_HUMAN	Q12794–2	Yes	Soluble	Inactive	Yes	[43]
	CHIT1_HUMAN	Q13231–3	Yes	Soluble	Inactive	ND	[48]
	ACMSD_HUMAN	Q8TDX5–2	Yes	Soluble	Inactive	Yes	[49]
(βα)_8_ with loop insertion or deletion	PLCB2_HUMAN	Q00722–2	Yes	Soluble	Active	Yes	[50]
	ADC_HUMAN	Q96A70–4	Yes^a^	ND	Inactive	Yes	[44]
(βα)_8_ with βα substitution	NPL_HUMAN	Q9BXD5–2	ND	ND	ND	Yes	[51]
(βα)_8_ with β-strand substitution	AK1BF_HUMAN	C9JRZ8–2	Yes	Soluble	Active	ND	[52]

a Reported by *in vitro* translation.

ND: Not determined.

From the sequence substitutions observed in splice variants, the β-strand substitution was the most common genetic event constituting 7.4% of all splice variants (Figure 2a). Splice variant C9JRZ8-2 has the substitution of the β1 of the barrel and this protein variant has been previously reported as a soluble and catalytically active enzyme (Table 2). However, functional and structural consequences of β-strand or α-helix substitutions have not been reported on protein members of the fold.

### β-strand and an α-helix substitutions in a model enzyme of the (βα)_8_ barrel fold

The Phosphorybosyl anthranylate isomerase (TrpF, enzyme, EC 5.3.1.24) from *E. coli*, a well-studied (βα)_8_ barrel involved in tryptophan biosynthesis, was selected as a scaffold for the substitution of the β-strand 7 and α-helix 3 (Figure S1). The β-strand and α-helix elements used as substitutions were selected from different structural positions of an enzyme not functionally related to the PRA isomerase activity of TrpF. These structural positions were chosen to avoid functional, structural, and evolutionary relationships, as previously observed in the substitution of βα-loops [25]. Thus, the α-helix 7 and β-strand 3 from the MetR enzyme (methyltetrahydrofolate, corrinoid iron-sulfur methyltransferase) were selected (Figure S1). In order to obtain folded and functional variants for further experimental analysis, we included the introduction of sequence diversity at the initial and terminal positions of each substitution as was previously suggested for βα-loops substitutions. Two libraries were constructed on the TrpF scaffold: a α-helix library that has the α-helix-7 of MetR replacing the original α-helix-3, and a β-strand library that has the β-strand-3 of MetR replacing the original β-strand-7 (Figure S1).

Because sequence diversity was introduced in both libraries, we analyzed the resulting abundance of folded and functional proteins after the application of corresponding selection pressures. The folded sequence space for the β-strand 7 library was higher than that of the α-helix 3 library: 80% and 39%, respectively (Figure S2). Despite the high number of folded variants observed in the β-strand library, only 4% of the total variants were functional, and these data are in agreement with the 6% observed in the α-helix library (Figure S2). Our results show that both types of secondary-structure substitutions can be tolerated by a (βα)_8_ barrel structure, although the original β-strand and α-helix elements that were replaced are located in conserved regions with different amino acid lengths compared to TrpF (Figure S1).

### Sequence analysis of functional variants and biochemical characterization of a chimeric variant

**Figure 3:**
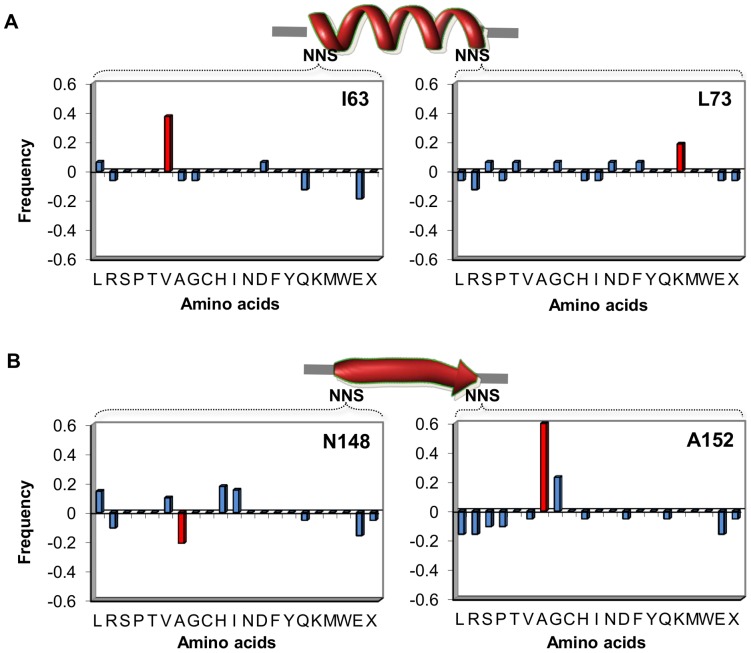
Sequence analysis under selective pressure at variable positions in chimera enzymes. Amino acids are represented by the one code letter. The histograms represent the relative frequencies of the selected versus unselected libraries. The red colored histograms (*) represent those amino acids at the variable positions that had a frequency either significantly higher (2 standard deviations) or lower than expected by chance. The sequence analysis for the α-helix 3 and β-strand 7 from MetR swapped into the TrpF scaffold are shown in A and B, respectively. The variable positions are represented by the NNS codon. The three-dimensional structure of the *E. coli* enzyme (PDB: 1PII) was used to identify the variable positions. The amino acid numbering of TrpF is according to gene reported in [25].

To investigate which amino acids were selected under functional constraints, we performed a sequence analysis of the variants complementing the PRA isomerase activity and compared those with the residue frequency observed without this selective pressure in each library. After normalizing the observed frequencies of each amino acid under selective versus nonselective pressure (Tables S7, S8, S9, and S10), we found a statistically significant overrepresentation of certain amino acids (Figure 3a and 3b). To compare the selected residues of the chimeric variants with the residues naturally found in the wild-type enzymes, we performed a structural alignment of the TrpF and MetR enzymes, and the sequence distribution observed at the corresponding randomized positions is shown in Figure S3. From this analysis, high sequence conservation for the positions preceding the swapped elements was observed (Figure S3). The amino acids found as over-represented in the functional chimeric TrpF variants (Figure 3a and 3b) are similar to the amino acids at this position in wild-type TrpF enzymes (Figure S3). In contrast, these amino acids are markedly different from the most conserved amino acid in the donor or host protein for the corresponding position. The selected residues in the variability positions of both β-strand and α-helix elements have a preference for the original residue in TrpF.

**Figure 4:**
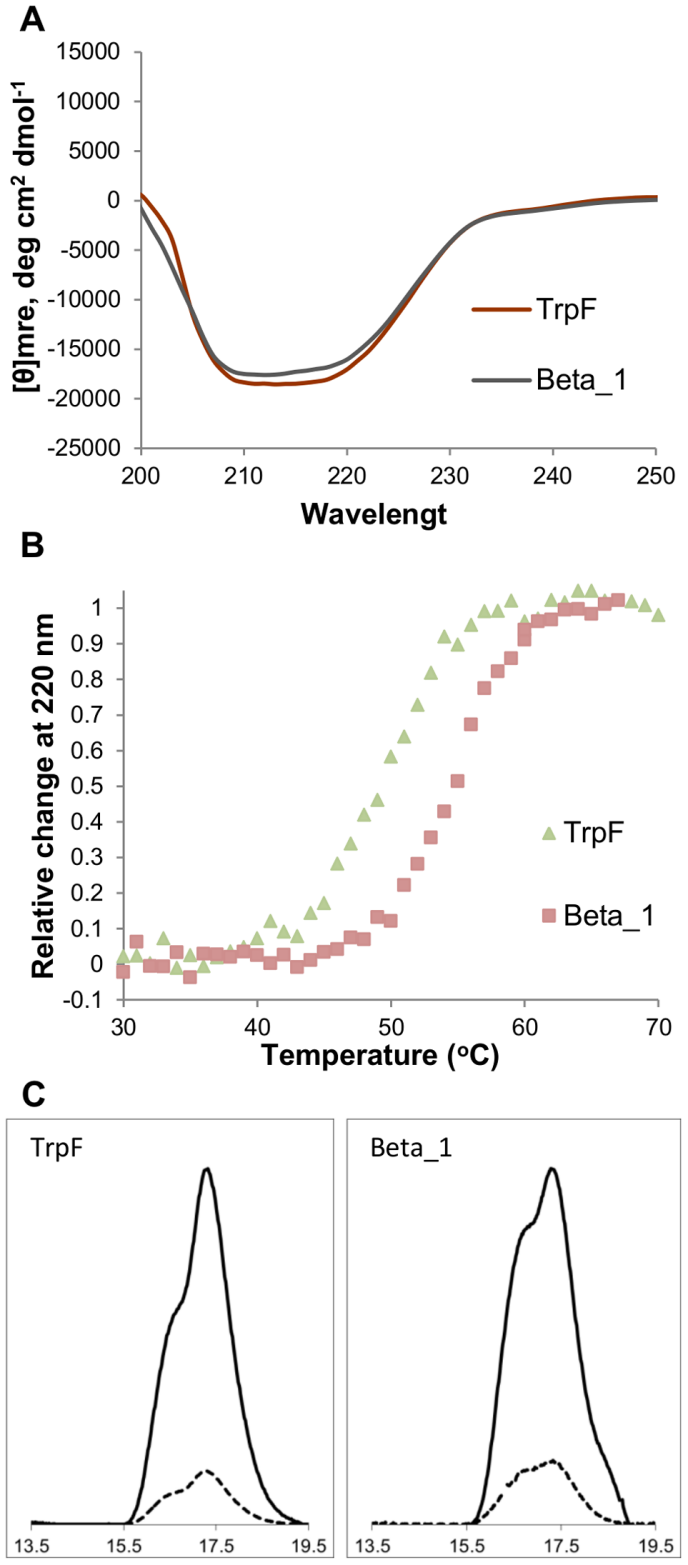
Structural analysis of variant Beta_1. The variant Beta_1 has a leucine residue in the N-terminal variable position and a glycine residue in the C-terminal variable position. A. Far-UV CD spectra. B. Thermal unfolding curves. C. Analytical gel-filtration chromatograms. A. The variant Beta1 showed only slight structural changes at the secondary structure level with respect to the wild-type enzyme. B. The variant Beta_1 (brown squares) has a T_m_ that is 5 degrees higher than the wt-TrpF enzyme (green triangles). C. The variant Beta_1 has an increase in the dimer population relative to the wild-type TrpF.

To analyze the functional and structural effects of β-strand or α-helix substitutions on the TrpF scaffold at the molecular level, we performed steady-state enzyme kinetics and structural studies in a chimeric variant that retained function. We selected a variant from the β-strand library because the replacement of an internal strand from a β-sheet can result in the loss of hydrogen bonds on both sides of the strand and requires the formation of several new ones to retain the native-like structure of the (βα)_8_ barrel. The analyzed variant, Beta_1, showed a 5-fold decrease in its k_cat_/K_m_ with respect to the PRA isomerase activity of the wild-type TrpF, largely due to a reduction in k_cat_ (Table 3). Hence, the modification of the original β-strand 7 can affect the correct conformation of the βα-loop 7. The far-UV CD spectra analysis suggests only slight structural changes at the secondary structure level as a consequence of the β-strand substitution relative to the wild-type enzyme (Figure 4a). A thermal denaturation curve shows that the Beta_1 variant forms a stable structure with an apparent thermal melting temperature (T_m_, _app_) of 55°C, which is 5 degrees higher than the T_m_ of the wild-type enzyme (Figure 4b and Table 3). This change in thermostability can be related to the increase of the dimer population of this variant (Figure 4c), as previously suggested for other mutants of the TrpF enzyme [25]. All the experimental analyses of the β-strand and α-helix substitution can improve our understanding of the biological role that these structural changes may be playing in protein evolution and in the functional expansion of the human proteome.

**Table 3:** Biochemical characterization of a chimeric variant.

Variant	k_cat_ (min^−1^)	K_m_ (µM)	k_cat_/K_m_ (M^−1^ s^−1^)	T_m,__app_ (°C) a
Beta_1	0.015	124.3	8.9	54.5
TrpF^b^	2028	28.9	1.2×10^6^	49.3

The overall standard errors of enzyme kinetic parameters are less than 20%.

a The apparent thermal melting temperature (°C).

b Data obtained from Ochoa-Leyva et al., 2011 [25].

## Discussion

On the functional level, splicing has been shown to contribute the structural and functional diversity of proteins [9]. The effects of alternative splicing on the novel functions of proteins range from changes in substrate or interacting partner specificity to the regulation of DNA-binding properties, the subcellular localization and the allosteric regulation sites in a target protein [9], [53], [54]. Alternative splicing can also influence the functional diversity at the level of protein expression [9], [54]. After extensive search of the biological literature for our splice variants, we found 15 splice variants with evidence for functional tissue-specific expression in the cell and 14 splice variants with evidence at protein level (Table 2). In addition, we identified clear evidence of stable protein products originating from such splice variants for the (βα)_7_ subdomain (Table 2). It has been previously suggested that variants Q13231-3 of gene CHIT1_HUMAN and Q8TDX5-2 of gene ACMSD_HUMAN might still be able to bind their substrates [48], [49]. The authors suggest that if this is the case, the metabolic relevance of the splice variant Q8TDX5-2 would be related to its capacity for binding and sequestering a reactive intermediate [49]. Variant P16278-2 of gene BGAL_HUMAN also termed EBP (elastin binding protein) in which half of the barrel was removed is an enzymatically inactive splice variant of lysosomal beta-galactosidase, but plays functional roles in the formation of extracellular elastic fibers (elastogenesis) and in the development of connective tissue [45]. Together, these observations suggest the existence of novel functional diversity for several splice variants of (βα)_8_ barrel proteins. It has been previously suggested that species-specific splice variants, relative to conserved splice variants, may less frequently play important functional roles [55]. In this regard, we found that the majority of splice variants have been conserved in more than two species (data not shown). We propose that these splice variants are more likely to maintain critical gene activities and they are not reflecting splicing noise or simply the result of selective pressure against insoluble proteins.

The results of our protein engineering experiments of β-strand substitution are consistent with the notion that alternatively spliced variants with β-strand substitutions, which was the main type of all the observed substitutions, can represent (βα)_8_ barrels that retain their original function. One example is the splice variant C9JRZ8-2 from AK1BF_HUMAN, where the original β1 of the barrel is substituted by another sequence, which was reported as an expressed, soluble, and active enzyme [52]. Moreover, alternative splicing allows a single gene to produce several splice variants which may affect several properties, such as structure, function, binding properties and stability of the encoded proteins [9]. For instance, we found that in the Beta_1 variant, the single β-strand substitution changes its stability, its catalytic activity and its capacity to oligomerize. Furthermore, the Beta_1 variant showed a 5-fold decrease in its k_cat_/K_m_ with respect to the PRA isomerase activity of the wild-type TrpF, largely due to a reduction in k_cat_ (Table 3). This work also reinforces the notion that DNA swapping of unrelated sequences can be relevant for the generation of molecular diversity [1], [2]. Although introducing diversity at both ends of the secondary-structure substitutions cannot be explained by alternative splicing events, one has to assume that extant proteins with functional splice variants were subject to some prior sequence variation during evolution. For instance, Tawfik and co-workers reported that compensatory substitutions may follow short insertions and deletions (InDels) accumulation [56]. The authors observed increased substitutions rates in the sequential vicinity of InDels. The primary assumption is that these correlated substitutions compensate for the deleterious effects of InDels and are therefore fixed by positive selection [56].

We found that the majority of splice variants are formed by (βα)_5_ and (βα)_7_ subdomains. Interestingly, the (βα)_5_ subdomain has been reported as an independently folding substructure in different barrel enzymes (Table 1). This subdomain can also maintain a significant amount of secondary structure and native-like tertiary conformation with a propensity to dimerize [28], [29]. Deviations from the canonical topology of the barrel structure have been reported. For example, quinolinic acid phosphoribosyltransferase, certain cellulases and flavoprotein contain only seven β-strands [38]–[40]. The existence of natural proteins containing the (βα)_7_ subdomain suggests that the splice variants resulting in this subdomain can retain a soluble and stable structure. The experimental existence of smaller substructures in the folding process and soluble and stable subdomains formed by (βα)_2_, (βα)_4_, (βα)_5_ or (βα)_6_ have been demonstrated in different (βα)_8_ barrel proteins [20]–[22], [28], [29]. Structural and functional resilience towards substitutions of βα subdomains and βα-loops have also been demonstrated in this fold [24]–[26], [32] which is in agreement with our observations of resilience to β-strand substitutions. Based on all of the experimental work surrounding diverse protein members of the (βα)_8_ barrel fold, we suggest that it is reasonable to hypothesize that a sizeable fraction of splice variants found in the human genome may represent structurally viable functional proteins.

**Figure 5:**
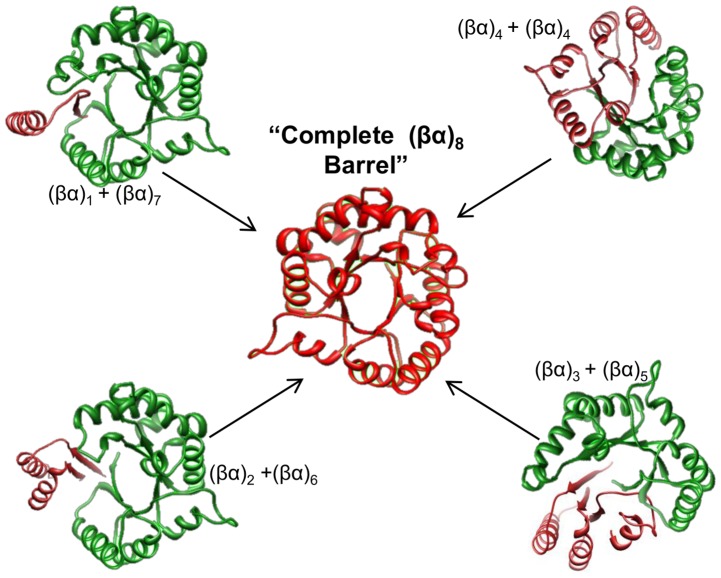
A model of structural assembly of novel (βα)_8_ barrels by splice variants. According to the experimental evidence of the existence of soluble and stable barrel subdomains in many different (βα)_8_ barrels, we suggest a process of subdomain assembly through genomic evolution which may result in multiple lineages of novel (βα)_8_ barrels in the human genome. In addition, the splice variants found in the human genome possibly form homo-dimers, heterodimers, or three-quarter barrel + quarter-barrel complexes to complete the (βα)_8_ barrel structure. The ribbon diagram shows a representation of the canonical (βα)_8_ barrel structure, and the different colors correspond to subdomains of different proteins that can be reassembled in the complete barrel (center). This model provides additional support for the proposed models on the origin of the (βα)_8_ barrel fold through the assembly of smaller subdomains. The 3D structure of PDB 1AW1 was used to illustrate the model.

Our results suggest that parts of the genes that encode different subdomains, such as the (βα)_2_, (βα)_4_ and (βα)_6_ part-barrels, can be fused, mixed, and matched through genomic evolution to yield new (βα)_8_ barrel proteins (Figure 5). This is in agreement with hypotheses on the origin of the (βα)_8_ barrel fold [22]. One hypothesis suggests that they have evolved by tandem duplication and fusion of an ancestral half-barrel [20]. The half-barrel can be formed by two rounds of gene duplication and fusion from a quarter-barrel ancestor [21]. An alternative evolutionary hypothesis suggests that three-quarter-barrels were viable evolutionary intermediates and that the combinatorial assembly of diverse pools of part-barrel subdomains gave rise to the many distinct lineages of (βα)_8_ barrel proteins [22]. Our analyses are consistent with both hypotheses, as they reveal many splice variants where a quarter-barrel, a half-barrel, and a three-quarter-barrel were created by alternative splicing events. In addition, we suggest that some of the splice variants function as homo-dimers or hetero-dimers to reconstruct the complete (βα)_8_ barrel *in vivo* (Figure 5). This notion is in agreement with the *in vivo* and *in vitro* subdomain reassembly previously observed for different barrel enzymes [23], [34], [57], including the reassembly of two half-barrels derived from different enzymes [58]. The expression of various alternative splice variants might offer an increased functional expansion of the proteome through combination of quarter-barrels, half-barrels, and three-quarter-barrels containing different functional sites in homodimeric or heterodimeric complexes (Figure 5). For instance, splice variants P16278-2, Q12794-3 and Q9BZP6-3, where one half of the barrel is removed, have been described to be expressed in a tissue-specific manner, and experimental validation of the existence of a stable protein product demonstrated for variant P16278-2 (Table 2).

Sequence diversity resulting from alternative splicing events is maintained in eukaryotic genomes throughout evolution. The human genome revealed a much smaller number of genes than anticipated, strengthening the notion that alternative splicing is a major factor in expanding protein diversity. The decade after the completion of the human genome sequencing has seen dramatic developments in sequencing technologies. These developments have generated a plethora of new sequences originating from alternative splicing events and have demonstrated that these events are not only involved in normal molecular processes but are also associated with human diseases such as cancer [59]–[61]. This work highlights the necessity of studying the folded and functional sequence space of alternatively spliced protein products in the laboratory and the importance of such studies in answering questions about the development and pathogenesis of many diseases where alternative splicing is involved. Our work suggests the possibility of crossing borders in sequence space without changing the natural robustness of a (βα)_8_ barrel and reinforces the hypothesis that (βα)_8_ barrel proteins appear to be considerably more tolerant to deletions, insertions, and replacements than previously stated.

## Materials and Methods

### Annotation of (βα)_8_ barrel proteins from human genome

The predicted (βα)_8_ barrel domain proteins encoded in *Homo sapiens* (taxon ID:9606) genome (Ensembl, GRCh37) were obtained using the fold recognition algorithms of the Gene3D program (v:11.0.0, CATH: v3.5.0) [30]. From the 22,111 protein-coding genes annotated for the human genome, 14,708 have at least one CATH Superfamily assignment. From these, 172 protein-coding genes were predicted with the CATH topology: 3.20.20, which corresponds to the (βα)_8_ barrel domain (Table S1). The splice variants for each gene were obtained from UniProtKB, and only the protein coding genes with experimental evidence that their splice variants exist at the mRNA level were selected (Table S2), resulting in 135 splice variants (Table S3). The existence at mRNA level of all the isoforms was also confirmed by searching in VEGA and ENSEMBL databases. The identification of the full-length protein sequence (canonical sequence) was extracted from the UniProtKB [31] database for each gene. The so-called “canonical sequence” is the most prevalent sequence and/or the most similar sequence among orthologous species [31]. The three types of splicing events (insertion, deletion and substitution) were identified after the sequence alignment of all splice variants for each gene, and all positional information annotated in the Table S4 and Tables S5 refers to the canonical sequence.

### Structures and models of the Full-Length (βα)_8_ barrels

The three-dimensional structures of the 70 full-length proteins were obtained from two sources. We first obtained the structures for the full-length proteins through mapping their amino acid sequences to the Protein Data Bank [62]. This searching returned PDB structures for 26 proteins. We then obtained structural models for the rest of the full-length proteins in our data set by searching their sequences in the MODBASE database [63], resulting in 44 structural models of high quality. All structural models were also optimized through energy minimization functions using the Chimera Package from UCSF [64] and through the Repair Object function of the FOLDX program [65], [66].

### Mapping spliced variants to the (βα)_8_ barrel structures

To identify the structural changes produced by the alternative splicing events in the original full-length proteins, each alternative splice variant was mapped to its corresponding protein structure, and the features of the regions being affected were analyzed and annotated manually using the Swiss-PDB viewer [67].

### Library design and construction of β-strand and α-helix substitutions

The two libraries were constructed using a previously reported strategy [25]. The diversity was generated by replacing the normal codon with an NNS codon at the respective positions and the replacements were performed using an overlap PCR strategy. Briefly, two oligonucleotides, which are partially complementary (12 bp), were designed for each secondary-structure replacement, one oligonucleotide corresponds to the noncoding DNA strand and the other oligonucleotide to the coding DNA strand. The coding oligonucleotides for α-helix and β-strand substitutions were 5′-CGGACCTTCCTTGCCATGGCCNNSTC-GCTGGCGGCAGTGCAA-3′ and 5′-TCGCTTGGCNNSATGATCNNSGGGGG-CTTAGGCGCAGAT-3′, respectively. The noncoding oligonucleotides were 5′-AAGGAAGGTCCGGTTAATCAAGGGNNSATCGTGATTGCGGAACAC-3′ and 5′-TAAGCCCCCNNSGATCATNNSGCCAAGCGATTGACCATT-3′, respectively. In all oligonucleotides, an NNS codon (underlined) that replaced the variability positions was introduced. Libraries were independently constructed by PCR and final products were ligated into the pDAN5 plasmid. The resulting libraries contained 10^5^ different variants that broadly covered the theoretical sequence diversity of 1024 variants for each library. Approximately 18 plasmids for each library were sequenced to confirm the corresponding β-strand or α-helix substitution and to analyze the statistical distribution of the sequence diversity introduced at both variable positions. From the sequence analysis of a pool of variants from each library, we can conclude that approximately 99% of the generated variants were correctly constructed and the sequence diversity introduced at both ends of the β-strand and α-helix elements is according to the sequence distribution for a NNS codon (data not shown).

### Assignment of folded and functional sequence spaces

Folded and functional sequence spaces were estimated using the strategy previously reported to analyze the Structure-Function Loop Adaptability in (βα)_8_ proteins [25]. Functional sequence space was calculated as the ratio of the number of variants that maintain the functional proficiency for PRA isomerase activity. To this end, the number of CFU (colony forming units) complementing the tryptophan auxotroph *E. coli* JM101ΔtrpF strain in M9 minimal media was measured and compared with the CFU grown without this functional selective pressure in LB medium. The libraries were previously fused to the CAT gene as an *in vivo* folding reporter. Folded sequence space was estimated according to the capacity of the variants to grow in the presence of chloramphenicol. Thus, the folding competence was calculated as the ratio of the number of CFU grown in LB amp plus chloramphenicol media (under folding selective pressure) to the number of CFU grown in LB amp media (without folding selective pressure).

### Sequence analysis of the functional sequence space

The sequences found in the variability positions from the variants growing under selective pressure for PRA isomerase activity (M9 minimal media) were compared with the sequences from the variants growing without this selective pressure (LB ampicillin). Sequences found in both conditions are illustrated in Tables S7, S8, S9, and S10. The amino acids observed in these positions were converted to frequencies and the difference between these indicates the discrepancy of occurrence for each residue between these two conditions (Figure 3). The average and standard deviation of the frequencies were used to determine, with 95% confidence, the negative or positive selection (red colored histograms in Figure 3) for specific amino acids in the corresponding variability positions.

### Biochemical and biophysical characterization of one variant

Expression and purification of variant Beta_1 was performed using the expression vector pET-28b (Stratagene), as previously described for wild-type TrpF [25]. Michaelis-Menten enzyme kinetics of PRA isomerase activity was determined using the protocol previously reported [25]. The kinetic parameters were obtained by fitting initial rates to the Michaelis–Menten model using nonlinear fit analysis with the public available program MicroCal Origin 5.0. The kinetic data shown in Table 3 represent the average of at least three independent experiments using freshly purified enzyme. The CD measurements were carried out using a J-715 spectropolarimeter (JASCO) equipped with a Peltier temperature control supplied by JASCO. The far-UV CD spectra were collected from 190 to 260 nm at 25°C in a 0.1-cm path length cell. Proteins were measured at a concentration of 0.3 mg/ml in 10 mM potassium phosphate buffer at pH 7.6, 1 mM EDTA and 1 mM beta- mercaptoethanol. Eight replicate spectra were collected from each sample to improve the signal-to-noise ratio. The thermal denaturation process was analyzed by measuring the change in ellipticity at 220 nm as a function of temperature, which was increased at a rate of 0.3°C min^−1^. The thermal denaturation curves were normalized assuming a linear temperature dependence of the baselines for native and denaturated states. The apparent thermal melting temperature (T_m, app_) was determined by identifying a midpoint temperature between the native form (linear interpolation of the native region) and the denatured form (the lowest point or linear interpolation of the unfolded region) on the thermal unfolding curves. The intermolecular associations were analyzed by size exclusion chromatography in an AKTA FPLC with a superose HR12 column (GE Healthcare). Purified protein in an initial volume of 0.15 ml was eluted at a flow rate of 0.4 ml min^−1^ on a Superdex 200 column (GE Healthcare) that was previously equilibrated in 50 mM HEPES buffer (pH 7.6) and 100 mM NaCl.

## Supporting Information

Figure S1 **Design of β-strand and α-helix substitutions in a model (βα)_8_ barrel enzyme.**
(DOCX)

Figure S2 **Theoretical, folded and functional sequence spaces for libraries secondary-structure substitutions.**
(DOCX)

Figure S3 **Sequence analysis found at variable positions in natural TrpF and MetR enzymes.**
(DOCX)

Table S1 **Number of protein coding genes from the human genome predicted with a (βα)_8_ barrel domain.**
(XLSX)

Table S2 **Full-length protein coding genes of (βα)_8_ barrels and number of experimentally comfirmed splice variants for each gene.**
(XLSX)

Table S3 **Experimentally reported splice variants of the 70 full length proteins.**
(XLSX)

Table S4 **Mapping of splicing variants into the structures of the full-length (βα)_8_ barrel proteins.**
(XLSX)

Table S5 **Sequence and structural details of the spliced variants with predicted structural changes affecting the (βα)_8_ barrel domain.**
(DOCX)

Table S6 **Superfamily assignment for the splice variants in which the structure of the (βα)_8_ barrel is affected.**
(DOCX)

Table S7 **Sequences found without selective pressure for β-strand library.**
(DOCX)

Table S8 **Sequences found under selective pressure for β-strand library.**
(DOCX)

Table S9 **Sequences found without selective pressure for α-helix library.**
(DOCX)

Table S10 **Sequences found under selective pressure for α-helix library.**
(DOCX)
