# Supplementary material for: Alternative Splice Variants in TIM Barrel Proteins from Human Genome Correlate with the Structural and Evolutionary Modularity of this Versatile Protein Fold
Source: PLoS One. 2013 Aug 12;8(8):e70582. doi: 10.1371/journal.pone.0070582 (PMC3741200; doi:10.1371/journal.pone.0070582)
Supplement: Figure S1 — Design of β-strand and α-helix substitutions in a model (βα)8 barrel enzyme. (DOCX) [file pone.0070582.s001.docx]

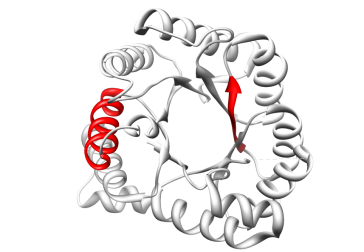

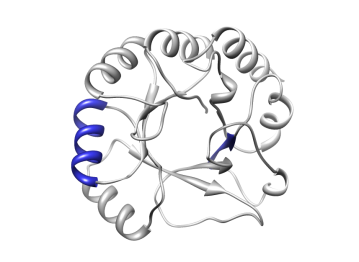

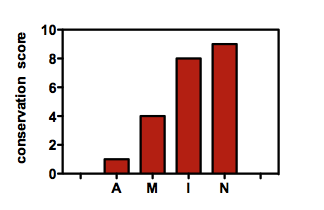


**a)**


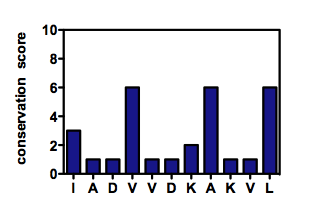

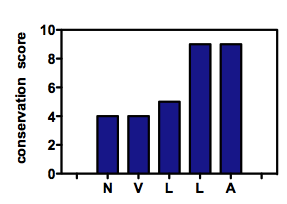


**α-helix-3**

**β-strand-7**


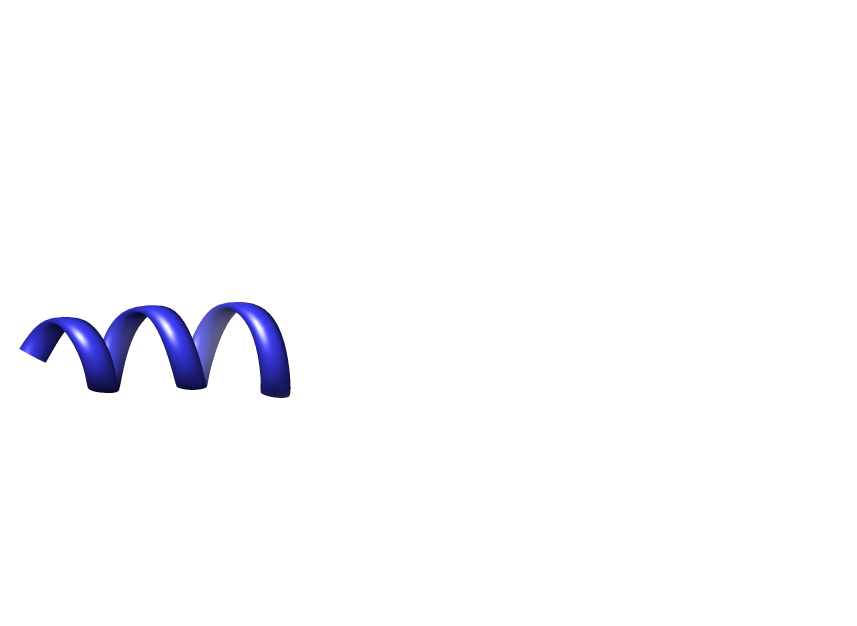

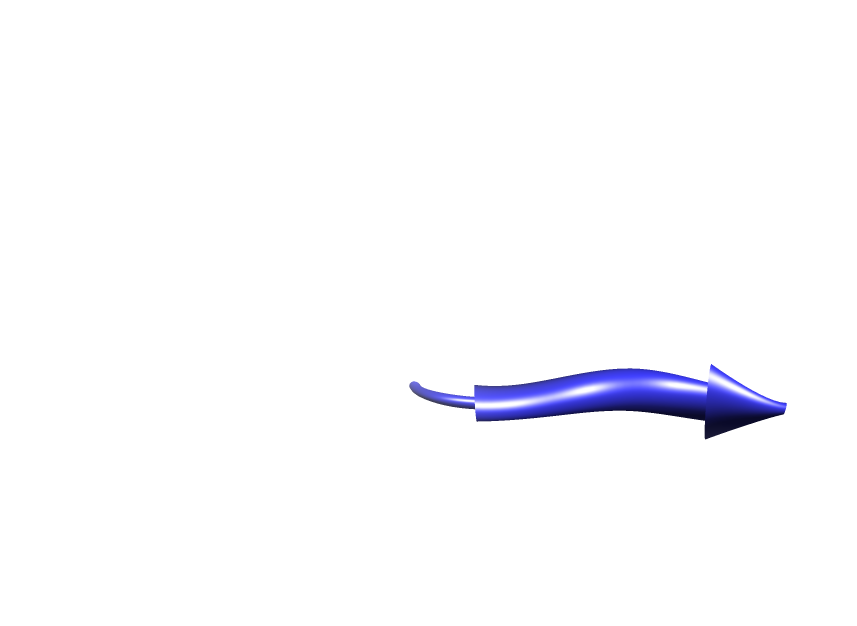


**b)**

**α-helix-7**

**β-strand-3**


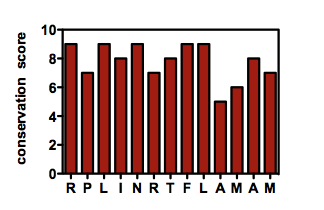

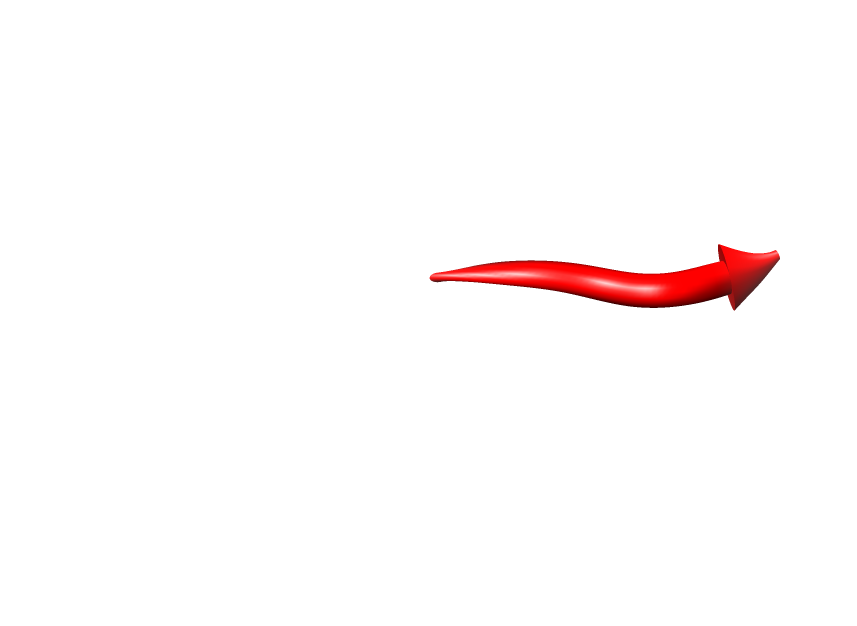

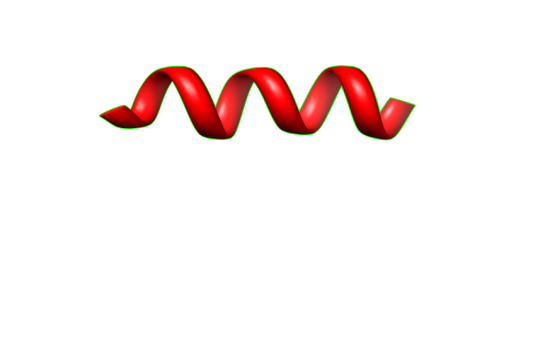


**Figure S1. Design of β-strand and α-helix substitutions in a model (βα)_8_ barrel enzyme.** Tertiary structures with a zoom representation of the β-strand and α-helix substitutions and their sequence conservation level are shown for the TrpF (A) and MetR (B) enzymes. The α-helix 7 and β-strand 3 of MetR (shown in red) were used to replace the α-helix 3 and β-strand 7 from TrpF (shown in blue), respectively. The β-strand and α-helix substitutions have different amino acid lengths. The 3D structures of TrpF (PDB: 1PII) and MetR (PDB: 1F6Y) were rendered using the Chimera package from UCSF [43]. A sequence conservation score for each amino acid of the secondary structure elements was calculated from a multiple sequence alignment of homologous enzymes through the ConSurf Server [47]. The amino acid numbering of TrpF is according to gene reported by Kirschner et al.
