# Supplementary material for: Alternative Splice Variants in TIM Barrel Proteins from Human Genome Correlate with the Structural and Evolutionary Modularity of this Versatile Protein Fold
Source: PLoS One. 2013 Aug 12;8(8):e70582. doi: 10.1371/journal.pone.0070582 (PMC3741200; doi:10.1371/journal.pone.0070582)
Supplement: Figure S2 — Theoretical, folded and functional sequence spaces for libraries secondary-structure substitutions. (DOCX) [file pone.0070582.s002.docx]

**
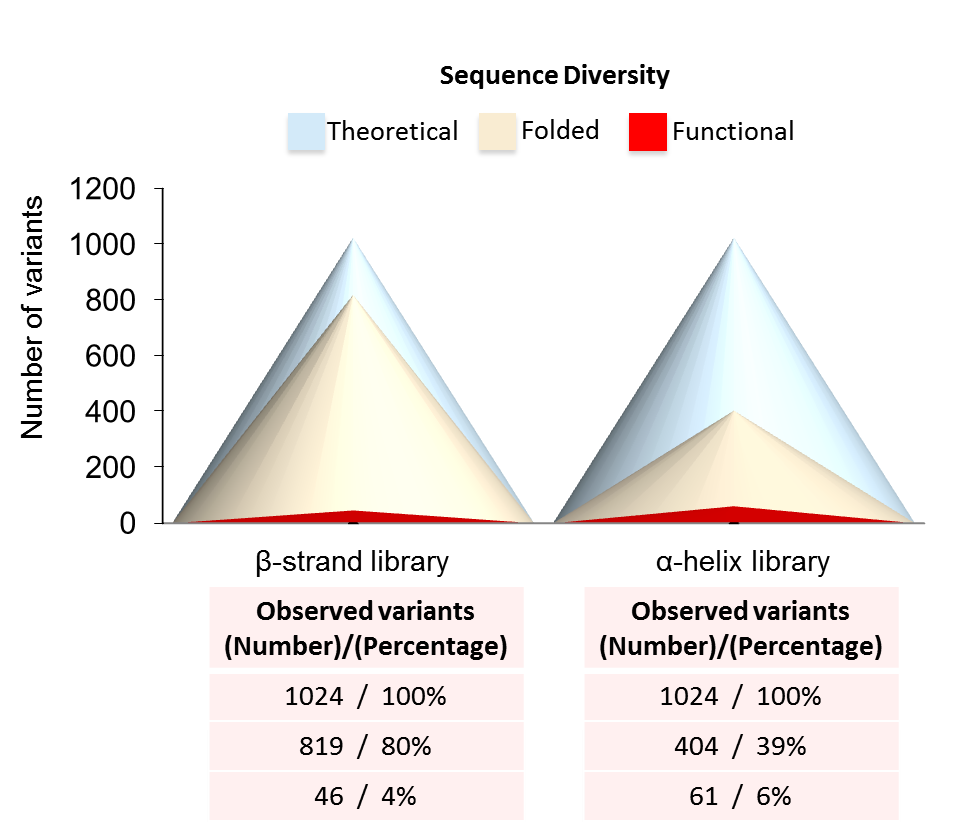
**

**Figure S2. Theoretical, folded and functional sequence spaces for libraries secondary-structure substitutions.** The theoretical sequence space for each library is 1024 variants because of the introduction of two NNS codons flanking the replaced secondary structure elements. The number and percentage of folded and functional variants were calculated as described in the materials and methods. The triangles represent the number/percentage of variants within each sequence space.
