# Supplementary material for: Alternative Splice Variants in TIM Barrel Proteins from Human Genome Correlate with the Structural and Evolutionary Modularity of this Versatile Protein Fold
Source: PLoS One. 2013 Aug 12;8(8):e70582. doi: 10.1371/journal.pone.0070582 (PMC3741200; doi:10.1371/journal.pone.0070582)
Supplement: Figure S3 — Sequence analysis found at variable positions in natural TrpF and MetR enzymes. (DOCX) [file pone.0070582.s003.docx]

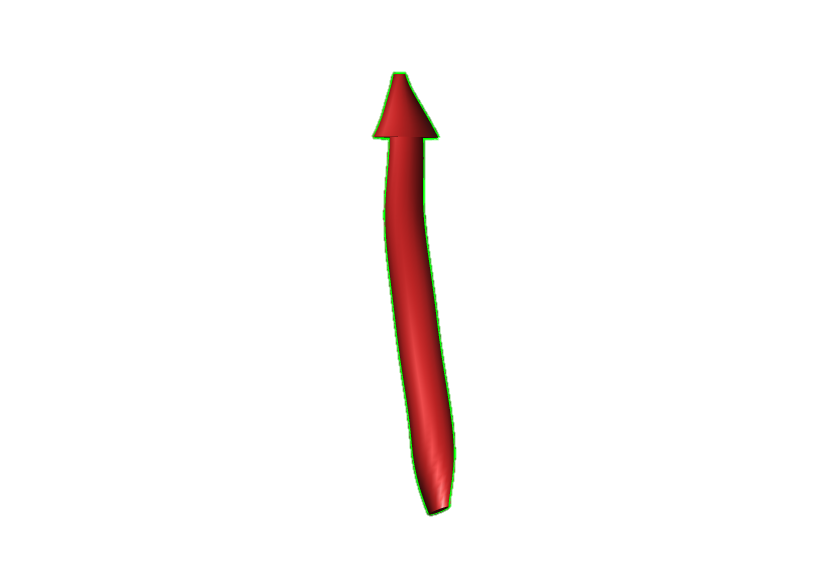

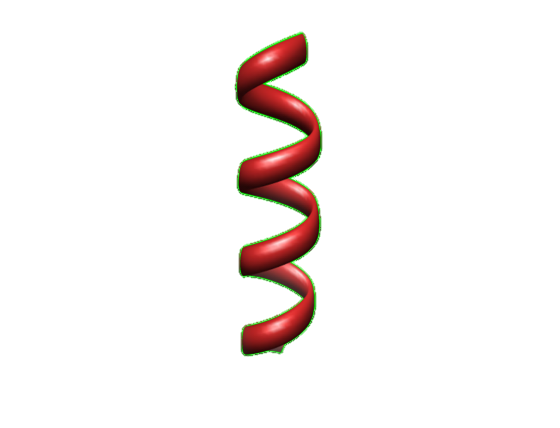


**I63**


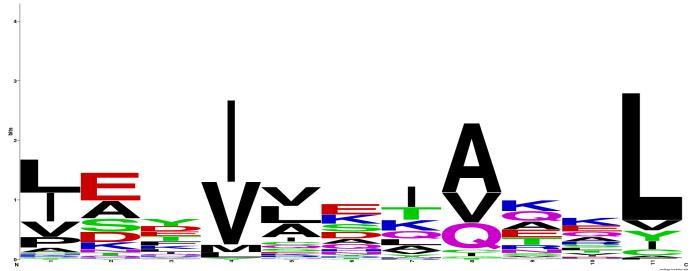


Bits

2

4

**L73**


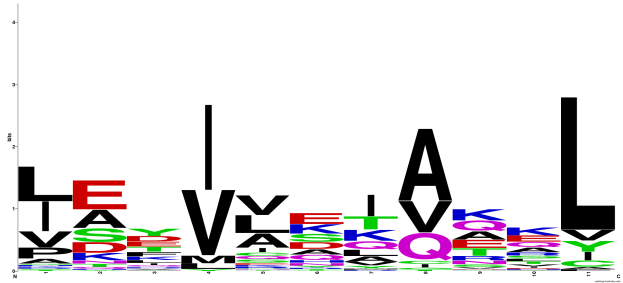


Bits

2

4

**R207**


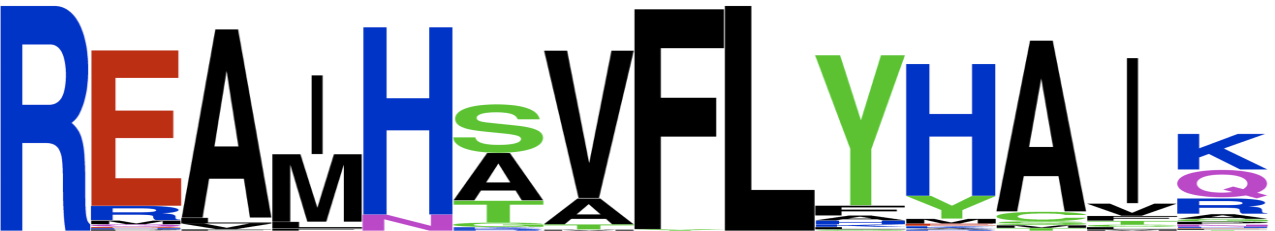


Bits

2

4


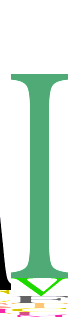


**M219**

Bits

2

4

**N148**


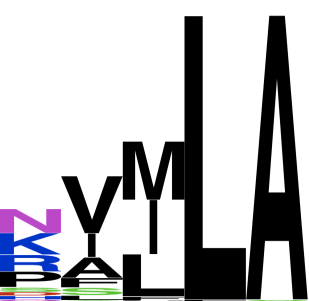


Bits

2

4

**A152**


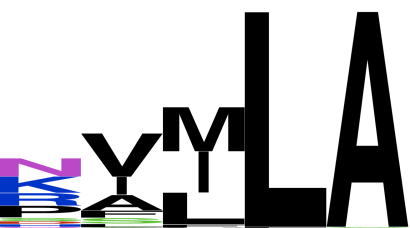


Bits

2

4

**A93**


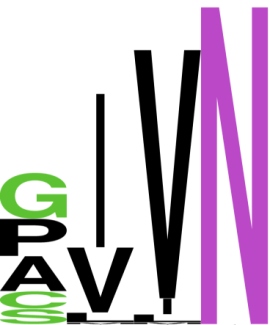


Bits

2

4

**N96**


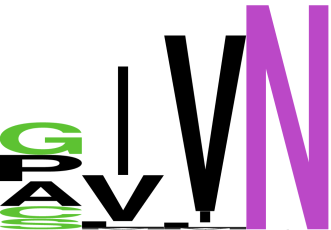


Bits

2

4

TrpF

MetR

**a)**

**b)**

TrpF

MetR

TrpF

MetR

TrpF

MetR

**Figure S3**. **Sequence analysis found at variable positions in natural TrpF and MetR enzymes**. The natural sequence diversity found at variable positions of β-strand and α-helix elements in the wild-type enzymes is shown for α-helix 3 of TrpF and α-helix 7 of MetR in a) and for β-strand 7 of TrpF and β-strand 3 of MetR in b). Sequence distribution was obtained from the multiple sequence alignments constructed in figure S1. The three-dimensional structure of the *E. coli* enzyme (PDB: 1PII) was used to identify the variable positions. The amino acid numbering of TrpF is according to gene reported by Kirschner et al.
