# Supplementary material for: Alternative Splice Variants in TIM Barrel Proteins from Human Genome Correlate with the Structural and Evolutionary Modularity of this Versatile Protein Fold
Source: PLoS One. 2013 Aug 12;8(8):e70582. doi: 10.1371/journal.pone.0070582 (PMC3741200; doi:10.1371/journal.pone.0070582)
Supplement: Table S5 — Sequence and structural details of the spliced variants with predicted structural changes affecting the (βα)8 barrel domain. (DOCX) [file pone.0070582.s008.docx]

**Table S5. Sequence and structural details of the spliced variants with predicted structural changes affecting the (βα)_8_ barrel domain**

| Protein ID | The sequence of the isoform differs from the canonical sequence as follows: | The structural changes of the isoform differs from the canonical structure as follows: | Description of the probable structure of the isoform |
| --- | --- | --- | --- |
| O43820-4 | Missing residues 1-249 and 299-328 | Missing βα(1-5)-loop-β6-loop(half) and α(7)-loop β8-loop | (βα)_1_ subdomain |
| Q86XE5-3 | Missing residues 71-233 | Missing βα(2-7) | (βα)_2_ subdomain |
| Q12794-5 | Missing residues 1-259 | Missing βα(1-5)-loop-β6-loop | (βα)_2_ subdomain |
| O43820-3 | Missing residues 1-249 | Missing βα(1-5)-loop-β6-loop(half) | (βα)_2_ subdomain |
| Q9H227-2 | Missing residues 95-401 | Missing α2-loop-βα(3-7) | (βα)_3_ subdomain |
| Q96A70-5 | Missing residues 94-251 | Missing α2-loop-βα(3-6)-loop- β7-loop | (βα)_3_ subdomain + other domain |
| Q9BXD5-5 | Exchanging residues 78-109 LDQVIIHVGALSLKESQELAQHAAEIGADGIA → SNHHKLGTIRTTQSRQSSFRRQLKAWHSGSHL and Missing residues 110-320 | Exchanging βα(3)-loop-β4(half) and missing β4(half)-loop-α4-loop-βα(5-8) | (βα)_3_ subdomain |
| P52895-2 | Exchanging residues 124-139: PGEEVIPKDENGKILF → EDIGILTWKKSPKHNS and Missing residues 140-323 | Exchanging the loop βα 4 by another sequence and missing loop(half)-α4-loop-βα(5-8) | (βα)_4_ subdomain |
| P16278-2 | Exchanging residues 83-244 YVPWNFHEPW...GLYTTVDFGT → LPGSCGQVVGSPSAQDEASPLSEWRASYNSA | Exchanging β2(two residues)-loop-α2-loop-βα(3-5)-loop-β6-loop(half) by 31 residues | (βα)_4_ subdomain + other domain |
| Q9BZP6-3 | Missing residues 1-161 | Missing βα(1-3)-loop-β4-loop-α4(half) | (βα)_4_ subdomain |
| O95620-2 | Missing residues 1-121 | Missing βα(1-3)-loop-β4-loop(half) | (βα)_4_ subdomain |
| Q12794-3 | Missing residues 1-182 | Missing βα(1-3)-loop-β4-loop-α4(half) | (βα)_4_ subdomain |
| A8MPS7-3 | Exchange of residues 111-136: EELEAQLSCFRELLGRAPTHADGHQH → SRSYRRMLARTPRAPPGGCGRSSRPN and Missing residues 137-323 | Exchange of α3(half)-loop-β4-loop(half) by other sequence and Missing loop(half)-α4-loop-βα(5-8) | (βα)_4_ subdomain |
| A8MPS7-2 | Exchange of residues 143-149: VCQVFAE → GQTPSWA and Missing residues 150-323 | Exchange of 7 residues of α4 by another sequence and missing α4(half)-loop-βα(5-8) | (βα)_4_ subdomain |
| Q5T013-2 | Missing residues 1-73 and 248-277 | Missing βα(1-2)-loop-β3-loop(half), and β8(half)-loop-α8 | (βα)_4_ subdomain |
| A6NNW6-2 | Missing residues 56-333 and 409-450 | Missing β1-loop-β2-loop-α1 and loop(half)-α3-loop-β4-loop | (βα)_5_ subdomain |
| Q5T013-4 | Missing residues 1-73 | Missing βα(1-2)-loop-β3-loop(half) | (βα)_5_ subdomain |
| Q96JD6-5 | Missing residues 154-251 | Missing α4(half)-loop-βα(5-6)-loop-β7-loop | (βα)_5_ subdomain |
| Q6UWU2-2 | Missing residues 150-239 | Missing α3-loop-βα(4-5)-loop-β6-loop(half) | (βα)_5_ subdomain + other domain |
| Q9BZP6-2 | Missing residues 1-108 | Missing βα(1-2)-loop-β3-loop(half) | (βα)_5_ subdomain |
| Q12794-4 | Missing residues 210-435 | Missing loop-α5-loop-βα(6-8) | (βα)_5_ subdomain |
| Q9H2M3-2 | Missing residues 87-150 | Missing α2-loop-βα(3)-loop-β4 | (βα)_6_ subdomain |
| Q86YW0-2 | Missing residues 46-238 | Missing βα(1-2) | (βα)_6_ subdomain + other domain |
| Q8N9F7-3 | Exchanging residues 266-314: DHLTARGIQVYIWVLNEEQEYKRAFDLGATGVMTDYPTKLRDFLHNFSA → EPLHPASKRNFEGHCSYLVVSCYF | Exchanging the α(half)-loop-βα(7-8) by 13 residues | (βα)_6_ subdomain |
| P51857-3 | Missing 153-193 | Missing the α4(half)-loop-βα(5)-loop-β6(half) | (βα)_6_ subdomain |
| Q96JD6-2 | Missing residues 194-250 | Missing β6(half)-loop-α6-loop-β7-loop | (βα)_6_ subdomain |
| Q96JD6-3 | Missing residues 194-250 and 308-320 | Missing β6(half)-loop-α6-loop-β7-loop | (βα)_6_ subdomain |
| P04062-3 | Missing residues 1-161, exchanging residues 422-423 LA → PS and Missing residues 425-536 | Missing βα(1)-loop-β2 and loop(half)-α8, and exchanging two residues of the final part of the barrel | (βα)_6_ subdomain |
| P35914-2 | Missing residues 117-187 | Missing loop-βα(4)-loop-β5-loop-α5(half) | (βα)_6_ subdomain |
| Q96G46-2 | Exchanging residues 464-482 : LHGRSREQRYTKLADWQYI → VGLGTPRALGPRGRPRVPS and missing residues 483-650 | Exchanging β6(half)-loop-α6 and missing loop-βα(7-8) | (βα)_6_ subdomain |
| P13929-2 | Missing residues 150-177 | Missing β1(half)-loop-β2-loop | (βα)_6_ subdomain |
| Q8N0X4-2 | Missing residues 147-180 | Missing loop-β5-loop | (βα)_7_ subdomain |
| P20839-2 | Missing residues 84-108 | Missing βα(2) | (βα)_7_ subdomain |
| Q9NZK5-2 | Missing residues 1-241 and exchanging residues 242-251 YMEIRARLLP → MDSLEWNWAL | Missing βα(1) and exchanging β2-loop(half) | (βα)_7_ subdomain |
| O75038-3 | Missing residues 706-741 | Missing βα(8) | (βα)_7_ subdomain |
| Q8N9F7-2 | Exchanging residues 276-290: YIWVLNEEQEYKRAF → SFWNDAFWKQHSSPV and Missing 291-314 | Exchanging β7(half)-loop-α7 by another sequence and missing loop- βα(8) | (βα)_7_ subdomain |
| Q6DHV7-2 | Exchanging residues 258-355 ELCLTSNVKS...NHLKPRVLHI → GKAWSFRSSR | Exchanging β7(half)-loop-α7-loop-βα(8) by 10 residues | (βα)_7_ subdomain |
| Q6DHV7-3 | Missing residues 185-211 | Missing loop(half)-α4-loop-β5-loop(half) | (βα)_7_ subdomain |
| Q01432-3 | Missing residues 652-767 | Missing loop-βα(8) | (βα)_7_ subdomain |
| Q6P1N9-2 | Missing residue 1-47 | Missing βα(1)-loop | (βα)_7_ subdomain |
| Q96BW5-2 | Missing residues 234-280 | Missing loop(half)-α6-loop-β7-loop-α7(half) | (βα)_7_ subdomain |
| Q9H4A9-2 | Missing residues 43-129 | Missing βα(1)-loop | (βα)_7_ subdomain |
| Q8NCI6-4 | Exchanging residues 293-313 RDKPLLIMEYWVGWFDRWGDK → GASIDAGLLSDASQAATSSSL and missing residues 314-653 | Exchanging loop(half)-β7-loop(half) and missing α7-loop-βα(8) | (βα)_7_ subdomain |
| Q13231-3 | Missing residues 344-372 | Missing α7(half)-loop-β8-loop | (βα)_7_ subdomain |
| Q9BWS9-3 | Missing residues 204-234 | Missing α4-loop-β5-loop(half) | (βα)_7_ subdomain |
| Q96AT9-2 | Missing residues 43-68 and inserting residues 114 K → KSCSVTQAEVQWHSQGPLQ | Missing loop-α2-loop-β3(half) and an insertion of 19 residues in the loop α4-β5 | (βα)_7_ subdomain |
| Q9BXD5-4 | Missing residues 203-246 and exchanging residues 260-320 | Missing α7-loop-βα(8) | (βα)_7_ subdomain |
| Q9NZB8-4 | Missing residues 1-87 | Missing β1-loop(half) | (βα)_7_ subdomain |
| Q9NZB8-7 | Missing residues 1-87 | Missing β1-loop(half) | (βα)_7_ subdomain |
| Q5T013-3 | Missing residues 248-277 | Missing β8(half)-loop-α8 | (βα)_7_ subdomain |
| Q12794-2 | Missing residues 301-330 | Missing α7-loop-β8-loop | (βα)_7_ subdomain |
| O43820-2 | Missing residues 299-328 | Missing α7-loop-β8-loop | (βα)_7_ subdomain |
| P08236-2 | Missing residues 305-355 | Missing β1-loop(half) | (βα)_7_ subdomain |
| Q8TDX5-2 | Missing residues 1-58 and exchanging residues 59-83 RIREMDQKGVTVQALSTVPVMFSYW → MGKSSEWCERIAGIQKFVLEKWTKK | Missing β1-loop and exchanging α1-loop-β2-loop | (βα)_7_ subdomain |
| Q9HCC8-3 | Insertion of 51 residues at position 436: K → KDRFLLPAQAGLKLLASSNLPASASQSAGITGLSHCPPQPPGYKHELSHLAM | Insertion of 51 residues in the loop α5-β6 | (βα)_8_ barrel with an insertion of 51 residues in the αβ loop |
| Q17R31-2 | Missing residue 201 | Missing one residue of the α4 | (βα)_8_ barrel with a deletion of 1 residue of the α4 |
| Q00722-2 | Missing residues 492-495 | Missing four residues of the loop βα(5) | (βα)_8_ barrel with a deletion of four residues in the βα loop |
| Q96A70-4 | Insertion of 12 residues at position 151 | Insertion of 12 residues in the loop α4-β5 | (βα)_8_ barrel with an insertion of 12 residues in the loop αβ |
| P20839-4 | Missing residues 104-108 | Missing α2(half) | (βα)_8_ barrel with a deletion of the half α2 |
| Q12794-7 | Exchanging residues 331-336: ESCQAI → VSLGLA and missing residues 337-435 | Exchanging α8(half) and missing α8(half) | (βα)_8_ barrel with a substitution of the α8 |
| P13716-2 | Exchanging residues 1-38 MQPQSVLHSG...SNLIYPIFVT → MPPTSSTPSL...QSISHPRSCR | Exchanging β1 | (βα)_8_ barrel with a substitution of the β1 |
| C9JRZ8-2 | Exchanging residues 1-50: MVLQMEPQVNSTNNFHQGPLDQPVGPLTGLKSSLLKDTTSAGPLLRPYPA → MATFVELSTKAKMPIVGLGTWR | Exchanging β1-loop(half) | (βα)_8_ barrel with a substitution of the β1 |
| P54803-4 | Exchanging residues 1-65 MAEWLLSASW...DGIGAVSGGG → MLGKSHGRAT...HQVTPEEKPA | Exchanging β1-loop(half) | (βα)_8_ barrel with a substitution of the β1 |
| P54803-5 | Exchanging residues 1-65 MAEWLLSASWQRRAKAMTAAAGSAGRAAVPLLLCALLAPGGAYVLDDSDGLGREFDGIGAVSGGG → MGFMVADLW and Missing residues 638-685 | Exchanging β1-loop(half) | (βα)_8_ barrel with a substitution of the β1 |
| Q0VAA5-2 | Exchanging of residues 289-303: SNRWNSHGPSLLSQE → RLALIPVYPLRFSR | Exchanging loop-β8 | (βα)_8_ barrel with a substitution of the β8 |
| Q9BXD5-2 | Exchanging residues 1-77 MAFPKKKLQG...EEWVTKGKDK → MSRAPGILAS...LTVTRLWAER | Exchanging βα(1-2) | (βα)_8_ barrel with a substitution of βα(1-2) |
| P54803-3 | Missing residues 66-88 | Missing loop(half)-α1-loop(half) | (βα)_8_ barrel with a deletion of the α1 |
