# Supplementary material for: Alternative Splice Variants in TIM Barrel Proteins from Human Genome Correlate with the Structural and Evolutionary Modularity of this Versatile Protein Fold
Source: PLoS One. 2013 Aug 12;8(8):e70582. doi: 10.1371/journal.pone.0070582 (PMC3741200; doi:10.1371/journal.pone.0070582)
Supplement: Table S6 — Superfamily assignment for the splice variants in which the structure of the (βα)8 barrel is affected. (DOCX) [file pone.0070582.s009.docx]

**Table S6. Superfamily assignment for the splicing variants in which the structure of the (βα)_8_ barrel is affected.**

| Protein ID | CATH superfamily |
| --- | --- |
| O43820-4 | Aldolase class I (3.20.20.70) |
| Q86XE5-3 | Aldolase class I (3.20.20.70) |
| Q12794-5 | Aldolase class I (3.20.20.70) |
| O43820-3 | Aldolase class I (3.20.20.70) |
| Q9H227-2 | Glycosidases  (3.20.20.80) |
| Q96A70-5 | Alanine racemase (3.20.20.10) |
| Q9BXD5-5 | Aldolase class I (3.20.20.70) |
| P52895-2 | NADP-dependent oxidoreductase (3.20.20.100) |
| P16278-2 | Glycosidases  (3.20.20.80) |
| Q9BZP6-3 | Glycosidases  (3.20.20.80) |
| O95620-2 | Aldolase class I (3.20.20.70) |
| Q12794-3 | Aldolase class I (3.20.20.70) |
| A8MPS7-3 | Glycoside hydrolase/deacetylase (3.20.20.370) |
| A8MPS7-2 | Glycoside hydrolase/deacetylase (3.20.20.370) |
| A6NNW6-2 | Enolase superfamily (3.20.20.120) |
| Q5T013-2 | Divalent-metal-dependent TIM barrel enzymes  (3.20.20.150) |
| Q5T013-4 | Divalent-metal-dependent TIM barrel enzymes  (3.20.20.150) |
| Q96JD6-5 | NADP-dependent oxidoreductase (3.20.20.100) |
| Q6UWU2-2 | Glycosidases  (3.20.20.80) |
| Q9BZP6-2 | Glycosidases  (3.20.20.80) |
| Q12794-4 | Aldolase class I (3.20.20.70) |
| Q9H2M3-2 | Not Assigned (3.20.20.330) |
| Q86YW0-2 | Phosphatidylinositol (PI) phosphodiesterase (3.20.20.190) |
| Q8N9F7-3 | Phosphatidylinositol (PI) phosphodiesterase (3.20.20.190) |
| P51857-3 | NADP-dependent oxidoreductase (3.20.20.100) |
| Q96JD6-2 | NADP-dependent oxidoreductase (3.20.20.100) |
| Q96JD6-3 | NADP-dependent oxidoreductase (3.20.20.100) |
| Q9NZK5-2 | Metal-dependent hydrolases  (3.20.20.140) |
| P04062-3 | Glycosidases  (3.20.20.80) |
| P20839-2 | Aldolase class I (3.20.20.70) |
| P35914-2 | Aldolase class I (3.20.20.70) |
| Q96G46-2 | Aldolase class I (3.20.20.70) |
| P13929-2 | Enolase superfamily (3.20.20.120) |
| Q8N0X4-2 | Phosphoenolpyruvate-binding domains (3.20.20.60) |
| O75038-3 | Phosphatidylinositol (PI) phosphodiesterase (3.20.20.190) |
| Q8N9F7-2 | Phosphatidylinositol (PI) phosphodiesterase (3.20.20.190) |
| Q6DHV7-2 | Metal-dependent hydrolases  (3.20.20.140) |
| Q6DHV7-3 | Metal-dependent hydrolases  (3.20.20.140) |
| Q01432-3 | Metal-dependent hydrolases  (3.20.20.140) |
| Q6P1N9-2 | Metal-dependent hydrolases  (3.20.20.140) |
| Q96BW5-2 | Metal-dependent hydrolases  (3.20.20.140) |
| Q9H4A9-2 | Metal-dependent hydrolases  (3.20.20.140) |
| Q8NCI6-4 | Glycosidases  (3.20.20.80) |
| Q13231-3 | Glycosidases  (3.20.20.80) |
| Q9BWS9-3 | Glycosidases  (3.20.20.80) |
| Q96AT9-2 | Aldolase class I (3.20.20.70) |
| Q9BXD5-4 | Aldolase class I (3.20.20.70) |
| Q9NZB8-4 | Aldolase class I (3.20.20.70) |
| Q9NZB8-7 | Aldolase class I (3.20.20.70) |
| Q12794-2 | Aldolase class I (3.20.20.70) |
| O43820-2 | Aldolase class I (3.20.20.70) |
| P08236-2 | Glycosidases  (3.20.20.80) |
| Q8TDX5-2 | Metal-dependent hydrolases  (3.20.20.140) |
| Q9HCC8-3 | Phosphatidylinositol (PI) phosphodiesterase (3.20.20.190) |
| Q17R31-2 | Metal-dependent hydrolases  (3.20.20.140) |
| Q00722-2 | Phosphatidylinositol (PI) phosphodiesterase (3.20.20.190) |
| Q96A70-4 | Alanine racemase (3.20.20.10) |
| P20839-4 | Aldolase class I (3.20.20.70) |
| Q12794-7 | Aldolase class I (3.20.20.70) |
| P13716-2 | Aldolase class I (3.20.20.70) |
| C9JRZ8-2 | NADP-dependent oxidoreductase (3.20.20.100) |
| P54803-4 | Glycosidases  (3.20.20.80) |
| P54803-5 | Glycosidases  (3.20.20.80) |
| P54803-3 | Glycosidases  (3.20.20.80) |
| Q0VAA5-2 | Phosphatidylinositol (PI) phosphodiesterase (3.20.20.190) |
| Q9BXD5-2 | Aldolase class I (3.20.20.70) |
| Q5T013-3 | Divalent-metal-dependent TIM barrel enzymes  (3.20.20.150) |

The CATH assignment corresponds to the full-length proteins of each splicing variant
