# Supplementary material for: Alternative Splice Variants in TIM Barrel Proteins from Human Genome Correlate with the Structural and Evolutionary Modularity of this Versatile Protein Fold
Source: PLoS One. 2013 Aug 12;8(8):e70582. doi: 10.1371/journal.pone.0070582 (PMC3741200; doi:10.1371/journal.pone.0070582)
Supplement: Table S7 — Sequences found without selective pressure for β-strand library. (DOCX) [file pone.0070582.s010.docx]

**Table S7.** Sequences found without selective pressure for β-strand library.

| Variants | **Amino position Carboxyl position**  **N148 A152** | |
| --- | --- | --- |
| 1 | A | E |
| 2 | Q | D |
| 3 | L | R |
| 4 | L | S |
| 5 | E | A |
| 6 | V | H |
| 7 | A | R |
| 8 | H | L |
| 9 | L | L |
| 10 | L | V |
| 11 | X | R |
| 12 | A | E |
| 13 | Q | L |
| 14 | A | P |
| 15 | R | X |
| 16 | L | S |
| 17 | L | P |
| 18 | R | Q |
| 19 | E | E |
