# Supplementary material for: Alternative Splice Variants in TIM Barrel Proteins from Human Genome Correlate with the Structural and Evolutionary Modularity of this Versatile Protein Fold
Source: PLoS One. 2013 Aug 12;8(8):e70582. doi: 10.1371/journal.pone.0070582 (PMC3741200; doi:10.1371/journal.pone.0070582)
Supplement: Table S8 — Sequences found under selective pressure for β-strand library. (DOCX) [file pone.0070582.s011.docx]

**Table S8.** Sequences found under selective pressure for β-strand library.

| Variants | **Amino position Carboxyl position**  **N148 A152** | |
| --- | --- | --- |
| 1 | L | A |
| 2 | L | A |
| 3 | H | G |
| 4 | H | A |
| 5 | L | G |
| 6 | I | A |
| 7 | I | G |
| 8 | L | A |
| 9 | L | A |
| 10 | V | A |
| 11 | V | A |
| 12 | H | A |
| 13 | L | A |
