# Supplementary material for: Alternative Splice Variants in TIM Barrel Proteins from Human Genome Correlate with the Structural and Evolutionary Modularity of this Versatile Protein Fold
Source: PLoS One. 2013 Aug 12;8(8):e70582. doi: 10.1371/journal.pone.0070582 (PMC3741200; doi:10.1371/journal.pone.0070582)
Supplement: Table S9 — Sequences found without selective pressure for α-helix library. (DOCX) [file pone.0070582.s012.docx]

**Table S9.** Sequences found without selective pressure for α-helix library.

| Variants | **Amino position Carboxyl position**  **I63 L73** | |
| --- | --- | --- |
| 1 | L | Q |
| 2 | Q | V |
| 3 | E | R |
| 4 | G | L |
| 5 | E | R |
| 6 | Q | I |
| 7 | R | E |
| 8 | A | P |
| 9 | G | T |
| 10 | A | T |
| 11 | V | X |
| 12 | L | P |
| 13 | P | R |
| 14 | E | R |
| 15 | L | E |
| 16 | V | H |
