# Supplementary material for: Alternative Splice Variants in TIM Barrel Proteins from Human Genome Correlate with the Structural and Evolutionary Modularity of this Versatile Protein Fold
Source: PLoS One. 2013 Aug 12;8(8):e70582. doi: 10.1371/journal.pone.0070582 (PMC3741200; doi:10.1371/journal.pone.0070582)
Supplement: Table S10 — Sequences found under selective pressure for α-helix library. (DOCX) [file pone.0070582.s013.docx]

**Table S10.** Sequences found under selective pressure for α-helix library.

| Variants | **Amino position Carboxyl position**  **I63 L73** | |
| --- | --- | --- |
| 1 | V | V |
| 2 | V | E |
| 3 | V | G |
| 4 | L | S |
| 5 | V | K |
| 6 | L | N |
| 7 | V | K |
| 8 | L | P |
| 9 | V | F |
| 10 | L | K |
| 11 | G | R |
| 12 | V | T |
| 13 | V | Q |
| 14 | A | T |
| 15 | D | R |
| 16 | P | T |
